# Autumn migration of the migrant hawker (*Aeshna mixta*) at the Baltic coast

**DOI:** 10.1186/s40462-023-00415-z

**Published:** 2023-08-24

**Authors:** Yvonne Oelmann, Diana Fiedler, Rune Michaelis, Meelis Leivits, Andreas Braun, Philipp Gschwind, Harald Neidhardt, Christoph Willigalla

**Affiliations:** 1 Geoecology, Department of Geosciences, University of Tübingen, 72070 Tübingen, Germany; 2 Lower Saxon Wadden Sea National Park Authority, 26382 Wilhelmshaven, Germany; 3 Estonian Environment Agency, Nigula Nature Centre, 86107, Reinu village, Estonia; 4 Geoinformatics, Department of Geosciences, University of Tübingen, 72070 Tübingen, Germany; 5 GÖG - Gruppe für ökologische Gutachten, 70599 Stuttgart, Germany; 6 Willigalla Ökologische Gutachten, 55124 Mainz, Germany

**Keywords:** Dragonfly, Migration, Bird observatory, Baltic coast, Stable isotopes, Hydrogen, Isoscape

## Abstract

**Background:**

Migratory insects are important for the provision of ecosystem services both at the origin and destination sites but – apart from some iconic species – the migration routes of many insect species have not been assessed. Coastlines serve as a funnel where migrating animals including insects accumulate. Migratory behaviour and captures of dragonflies in bird traps suggest autumn migration of dragonflies along coastlines while the origin and regularity of this migration remain unclear.

**Methods:**

Dragonfly species were caught at the bird observatory Kabli at the Baltic coast in Estonia in 2009, 2010 and 2015. For the 2015 data set, we used a stable hydrogen (H) approach to trace the potential natal origin of the migrant hawker (*Aeshna mixta*).

**Results:**

1079 (2009), 701 (2010) and 88 (2015) *A. mixta* individuals were caught during the study periods (35, 37 and 11 days in 2009, 2010 and 2015, respectively). The migration period lasted from end of August to end of September. Based on the results from our stable isotope analysis, we identified two populations of *A. mixta*: One (range of isotope signatures of non-exchangeable H [δ^2^H_n wing_]: −78‰ to −112‰) had a local likely origin while the other (δ^2^H_n wing_: −113‰ to −147‰) migrated from northerly directions even in headwind from the South. The former showed an even sex ratio whereas the actively migrating population was dominated by males.

**Conclusions:**

Our results suggest a regular southbound autumn migration of *A. mixta* along the Baltic coast. However, nearly half of the sampled individuals originated from the surroundings suggesting either no, partial or “leap-frog” migration. Contrary to our expectation, *A. mixta* did not select favourable wind conditions but continued the southbound autumn migration in the flight boundary layer even in case of headwinds. The dominance of males might indicate migration as a result of competition for resources. Further repeated, large-scale studies along the Baltic coast are necessary to pinpoint the migratory pattern and the reason for migration of *A. mixta*. Such studies should also comprise locations north of the known species range of *A. mixta* because of the rapid climate-change induced range expansion.

**Supplementary Information:**

The online version contains supplementary material available at 10.1186/s40462-023-00415-z.

## Introduction

Many species in the animal kingdom migrate across large geographical scales and thus, connect ecosystems [1–3]. Prominent examples of long-distance and partly multi-generational insect migration include butterflies (*Danaus plexippus* [4]; *Vanessa cardui* [5]) and dragonflies (*Pantala flavescens* [2, 6, 7]; *Anax junius* [8]). Migratory insects are important for the provision of ecosystem services both at the origin and destination sites [3, 9, 10], yet, migration routes of many insect species remain elusive.

One reason is linked to the challenge of tracking insect migration since common approaches are difficult to apply due to the small body size of the individuals and due to the fact that recapture is almost impossible [11, 12]. Animal tissue such as bird feathers or insect wings preserve the stable isotope signatures of for example carbon (C), nitrogen (N) and hydrogen (H) indicative of food and water sources [13]. The H isotope signature of C-bonded H in insect wings (δ^2^H_n wing_) is used as a marker for the natal origins [11]. Migration away from the natal origin can be tracked based on the comparison between the observed δ^2^H_n wing_ values of an individual against δ^2^H_n wing_ values to be expected in case of non-migrating namely sedentary individuals at the collection location. The latter is derived from the relationship between δ^2^H_n wing_ values of sedentary species and δ^2^H values of precipitation (δ^2^H_p_) that vary geographically [13]. Accordingly, so-called ‘wing isoscapes’ resulting from the relationship between δ^2^H_n wing_ values of sedentary species and δ^2^H_p_ were developed for several insect groups in North America [11, 14, 15]. Much less information on δ^2^H_n wing_ values of insects is available for Europe (but see studies on hoverflies [16] and moths [12]). Therefore, it is difficult to directly track the migration of e.g., dragonflies in Europe.

Coastlines serve as a funnel where migrating animals including dragonflies accumulate particularly during autumn [17–19]. In line, large numbers of dragonfly individuals have been caught in bird traps along migration routes at the Baltic coast in autumn [20–23]. Based on the increased numbers of individuals caught during Northerlies (a wind from the North) that are common at the Baltic coast in autumn, some authors attributed the capture of dragonfly individuals to wind drift rather than to migration [20]. For one of these species, namely *Aeshna mixta* (Latreille, 1805), massive and irruptive migration was assumed [19, 24]. Similarly, Knoblauch [17] suspected migratory behaviour of *A. mixta* from active southward orientation irrespective of wind direction at the Baltic coast in Latvia. They found that *A. mixta* selected favourable tailwinds for migration [17] which is assumed as an adaptation of insects to maximise the distance covered and to reduce energy costs [25, 26]. However, whether the autumn migration along the Baltic coast is a regular feature in the life cycle of *A. mixta* remains unclear.

The majority of migrating insect species follow the availability of resources or flee from unfavourable climate conditions, predation, parasitism and/or pathogen pressure [1, 2]. Ultimately, the migration destination – or even stops en route [18, 27, 28] – provide more favourable conditions for reproduction and hibernation [1]. Environmental conditions such as temperature changes are a cue to initiate the migration. Consequently, either hatching followed by spring migration to reproduction sites or hatching in spring or summer followed by autumn migration to reproduction or hibernation sites is common for many dragonfly species [1, 8, 18, 27, 29]. However, reproduction at the destination does not always seem to be the driver of migration [30, 31]. For dragonflies and other insect groups, it has been shown that large population densities towards the end of the life cycle might drive particularly males to migrate [24, 32] and avoid competition for resources by migration [1]. Therefore, more research is required to shed light on the drivers of dragonfly migration in autumn when the life cycle of most species ends [33].

In autumn, we captured *A. mixta* individuals using a Heligoland trap set up at a bird observatory in Estonia. Our study focused on analysing the migration of *A. mixta* by determining potential natal origins via stable isotope analysis of nonexchangeable H in wings. Our hypotheses are (i) that a dragonfly species, *A. mixta* sampled at a bird observatory in Estonia originates from northern locations and thus, proves southbound autumn migration of dragonflies at the Baltic coast, (ii) that migrating *A. mixta* individuals are preferentially caught in case of tailwind conditions namely Northerlies and that (iii) more males than females of *A. mixta* migrate along the Baltic coast during autumn.

## Materials and methods

### Study site, weather conditions and sample collection

In autumn each year, a Heligoland trap located approximately 150 m from the Baltic Sea shore is erected at the Kabli bird observatory, Estonia (58°0’51’’N 24°26’58’’E), to catch migrating birds. The trap stretches from the North to the South with a north facing entrance (Fig. S1). Dragonfly individuals were caught in autumn 2009 (22.08.-25.09.), 2010 (20.08.-01.10.) and 2015 (27.08.-06.09.). Throughout the study periods, the Heligoland trap was examined at hourly intervals (from 8 am to 7 pm) each day to determine the presence of dragonfly species. All dragonfly individuals present were identified to species level and sexed. The dragonfly individuals caught in the years 2009 and 2010 were released back into their natural habitat, while those collected in 2015 were euthanised by chloroform and preserved in plastic bags for subsequent analyses. In this study, we included only the individuals of *A. mixta* caught in 2015 (n = 88). We noted the colouration of the individuals (immature versus sexually mature). We measured the length of all four wings and cut half of the smaller hindwing of each *A. mixta* individual for isotope analysis (see below).

Wind directions recorded at the weather station in Jaagupi harbour (located 5 km north of Kabli) were available for the study periods in 2009 and 2010. Specifically, wind directions were documented at hourly intervals corresponding to the capture times of *A. mixta* individuals. However, for the study period in 2015, missing data for certain dates necessitated the use of supplementary information obtained online (Data from MET Norway; https://www.yr.no/en/details/table/2-591805/Estonia/P%C3%A4rnumaa/P%C3%A4rnu%20linn/Kabli). Data on temperature and wind speed during the study periods in 2009, 2010 and 2015 are provided in the supplementary information (Table S1).

For the years 2009 and 2010 with consistent data sets, we investigated the impact of wind directions on the abundance of captured dragonfly individuals. Each day, we counted the individuals caught under the same wind direction. If the wind direction changed during a day, we calculated separate sums for the different wind directions. Subsequently, we standardised the daily count of individuals for each wind direction by dividing it by the total number of hours that the respective wind direction prevailed during the entire capture period on that day. This normalisation approach enabled us to estimate the number of individuals caught per wind direction per day, irrespective of the frequency of occurrence of each particular wind direction.

### Life cycle of *Aeshna mixta*

The dragonfly species studied is the migrant hawker (*Aeshna mixta*). Oviposition by *A. mixta* takes place in fall and the eggs hibernate [33]. The larvae hatch at the end of March in Central and Northern Europe and adults emerge between the end of July and the end of September [34]. In contrast to other representatives of the Aeshnidae, *A. mixta* is an univoltine species, so the development from egg to adult takes only one year [35]. The main flight period in Northern Europe is from August to mid of September [33]. *A. mixta* is conspicuous among the Aeshnidae for its late flight period, which in Northern Europe is in the late summer [34, 36]. The dragonfly species is widespread and, currently, is expanding its range to the north [36]. In Sweden, the species has spread 300 km northward during just one decade [34].

### Stable isotope analysis

We used a steam equilibration procedure to account for the exchangeable proportion of H and calculate the δ^2^H values of non-exchangeable H [37, 38]. After the steam equilibration, stable hydrogen isotope ratios of dragonfly wings were measured with a TC/EA-IRMS device (vario PYRO Cube and IsoPrime 100, Elementar Analysesysteme GmbH, Germany). Further information on stable isotope analysis is provided in the supplementary information.

### Statistics, dragonfly wing isoscape and assignment of natal origins of dragonflies

Statistical analyses were performed in R Studio, version 4.0.3 [39]. To determine if the sampled individuals belonged to a single population, K-means clustering was employed. The goal was to test the existence of a single cluster that would encompass all individuals. The identification of distinct clusters was based on the Euclidean distance between the data and the clusters. This process involved iteratively moving data points between clusters until the minimum within-cluster sum of squares was achieved. If different clusters were present, a threshold could be established by comparing the two individuals with the most similar δ^2^H_n wing_ values, despite belonging to separate clusters.

Differences in the standardised number of *A. mixta* individuals caught per day among wind directions were tested using the Kruskal-Wallis test.

**Fig. 1:**
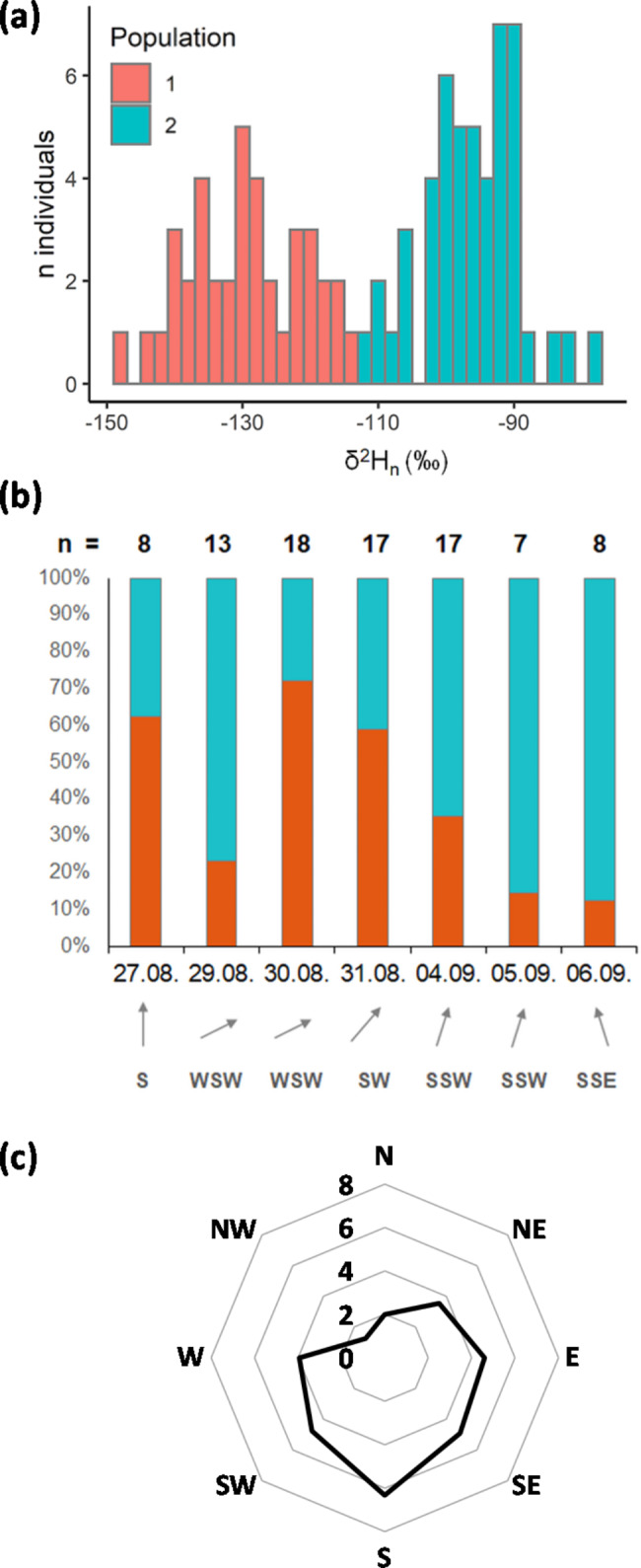
**(a)** Frequency distribution of δ^2^H_n wing_ values. **(b)** Contribution of the two populations (2015). **(c)** Caught individuals and wind directions (2009, 2010). In **(b)**, the contribution of the two populations at each collection day was expressed as percentage of the total caught individuals and the prevailing wind direction is depicted. The relationship between caught individuals and wind directions in **(c)** is provided as an average of the sum of individuals caught per day standardised to the number of hours the respective wind direction prevailed during the collection period at that day

**Fig. 2:**
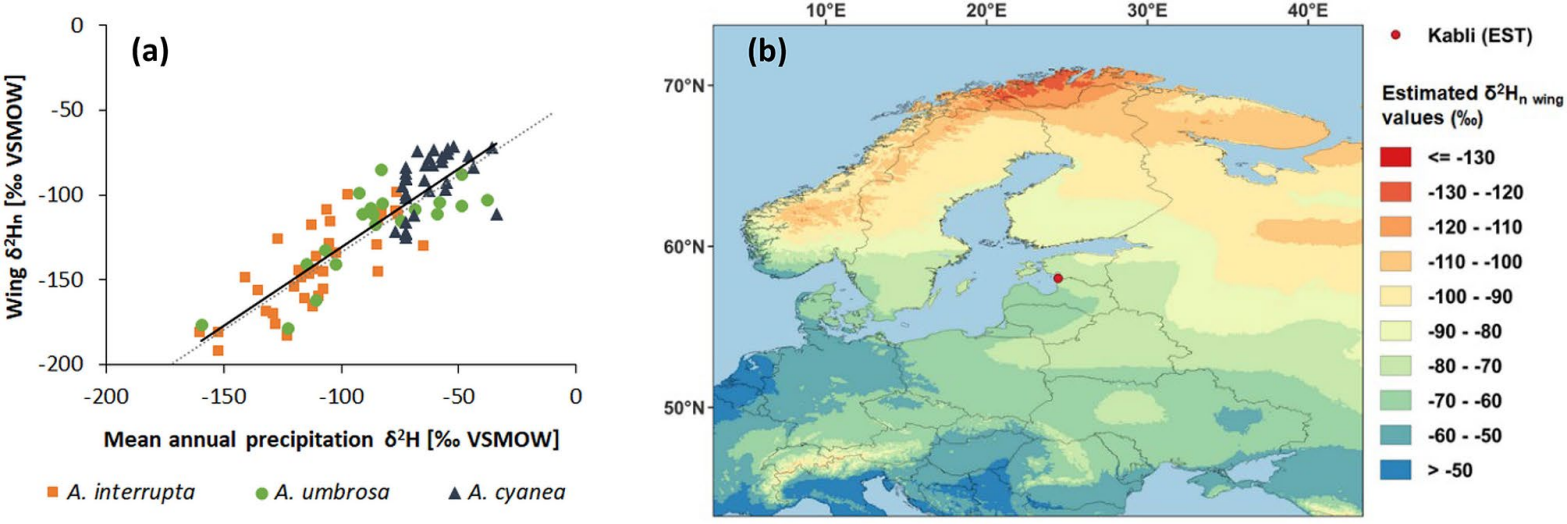
**(a)** Relationship between δ^2^H_p_ and δ^2^H_n wing_values and **(b)** the resulting European dragonfly wing isoscape. δ^2^H_n wing_ values of *Aeshna cyanea* (n = 34, black triangles) are from this study. Data on *Aeshna interrupta* (orange squares), *Aeshna umbrosa* (green circles) were taken from [11]. We used δ^2^H_p_ values of mean annual precipitation obtained using the ‘Online Isotopes in Precipitation Calculator’ [40–42]

**Fig. 3:**
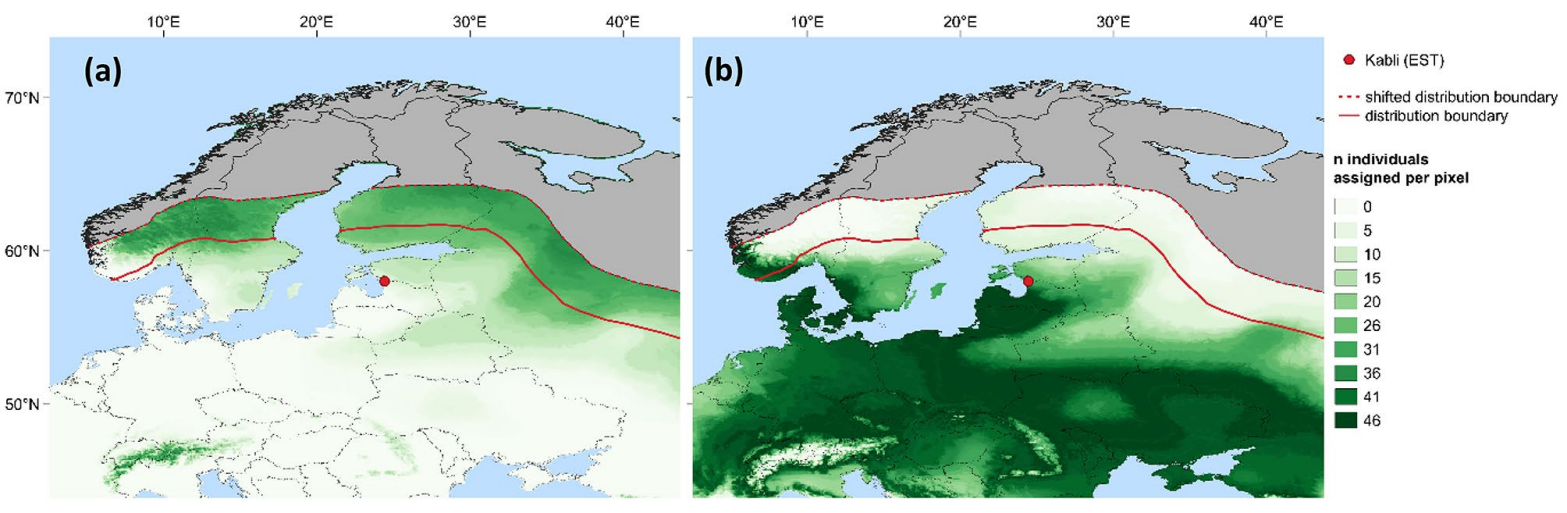
Probable origin of *Aeshna mixta* individuals caught at Kabli, Estonia. **(a)** and **(b)** refer to Population 1 and 2, respectively, caught at the Kabli bird observatory (58°0’51’’N 24°26’58’’E; red dot) between 27.08. and 06.09.2015. The hue indicates the number of individuals assigned to each pixel. The area of the probable origin was restricted to the area range of *A. mixta* [46] (solid red line) that was shifted 300 km northwards (dashed red line). The administrative boundaries follow Runfola, Anderson [60]

The relationship between δ^2^H_P_ and δ^2^H_n wing_ of sedentary species built the basis to set up a dragonfly wing isoscape. We collected a data set on δ^2^H_n wing_ values of the sedentary species *Aeshna cyanea* (Müller, 1764) across Europe (n = 34; Bosco-Fontana, Mantua, Pollino, Termoli (Italy); Norwich, Sudbury-Hill (Great Britain); Falsterbo (Sweden); Mtskheta-Mtianeti (Georgia); Kaiserslautern, Mainz, Schwäbisch Hall, Tübingen, Wiesbaden (Germany); Antonin, Borowice, Chalin, Kamień, Płocicz, Stankowo (Poland); Kabli bird observatory (Estonia)). The δ^2^H_p_ values of the locations where these species were collected was estimated using the ‘Online Isotopes in Precipitation Calculator’ [40–42]. Because these δ^2^H_p_ values only reach -76.8‰, we had to use an additional data set to include potential natal origins north of the study site in Estonia. To the best of our knowledge, no δ^2^H_n wing_ values of other sedentary dragonfly species in Europe are available. Therefore, we relied on δ^2^H_n wing_ values of sedentary Aeshnidae in North America (*Aeshna interrupta* (Walker, 1904), *Aeshna umbrosa* (Walker, 1908)) provided in [11]. We used the programme Web plot digitizer (Vers. 4.1) to convert Fig. 3 of Hobson, Soto [11] into data points (δ^2^H_p_ and dragonfly δ^2^H_n wing_). We then applied spatially explicit probability calculations [43] to evaluate the potential natal origin of each sampled dragonfly individual. Further details on the procedure can be found in the supplementary information. Using an arbitrary odds ratio of 2:1, we created binary raster maps (probability ≥ 66% = probable origin, binary pixel value coded 1; probability < 66% = not a probable origin, binary pixel value coded 0) for each individual. We produced a map by stacking the individual-specific binary pixel maps. At the extreme ends, a pixel in such a map could have a value of 0 indicating that this pixel was not assigned as a probable origin for any of the individuals or a value identical to the sample size meaning that this pixel was a probable origin of all of the individuals.

## Results

In all study years, southerly and southwesterly winds predominated (Fig. 1) with an average wind speed of 23 km h^− 1^ at the days where dragonflies were sampled in 2015 (Table S1). In 2009 and 2010, 1079 and 701 *A. mixta* individuals were caught, respectively. In 2015, 88 individuals of *A. mixta* were sampled. The sampled animals were characterised by sexually mature colouration, but did not show any bleaching due to age. The wings were hardened, showing partial signs of use (small rips along the wing edges). The first individuals were caught on 22.08., 20.08. and 27.08. and the last ones on 25.09., 01.10. and 06.09. in 2009, 2010 and 2015, respectively. In 2009, there seemed to be a peak in the number of caught individuals (> 40 individuals per day caught between 26.08. and 09.09.) while a peak was not evident in 2010 and 2015 (Fig. S2). The largest number of individuals caught during one day was 135 (2009), 94 (2010) and 18 (2015). In 2009 and 2010, there was a significant relationship between number of individuals caught and southerly and southwesterly wind direction (p < 0.04, Fig. 1c).

δ^2^H_n wing_ values of dragonfly individuals ranged from -78‰ to -147‰ (Fig. 1a). The frequency of δ^2^H_n wing_ values revealed two peaks indicating two populations with different origins. Subsequent k-means clustering divided individuals based on the distribution of their δ^2^H_n wing_ values into populations with δ^2^H_n wing_ values ranging from -113‰ to -147‰ (Population 1) and from -78‰ to -112‰ (Population 2; Fig. 1a). Population 1 contained 39 individuals and was dominated by males (64%) whereas Population 2 consisted of 49 individuals with an equal share of gender (49% male, 51% female). There were no obvious morphological differences (colouring, wing length) between the two populations (p > 0.05). The number of sampled individuals of both populations remained relatively stable (e.g. Population 2: 27.08.: 3, 29.08.: 10, 30.08.: 5, 31.08.: 7, 04.09.: 11, 05.09.: 6, 06.09.: 7; Fig. 1b).

We found a highly consistent relationship between the H isotope signatures of annual precipitation and of dragonfly wings by combining *A. cyanea* wings collected in Europe with those of different species of Aeshnidae from North America (Fig. 2a; δ^2^H_n wing_ = 0.93 ⋅ δ^2^H_p_ – 38.33, r = 0.86, p < 0.001). The regression parameters were then used to create a dragonfly wing isoscape for Europe (Fig. 2b) which served as a basis for the assignment of potential natal origins of sampled *A. mixta* individuals. Based on this, Population 1 could have migrated from northerly and easterly directions in Russia (Fig. 3a). For the majority of individuals (72%) with a probable natal origin in Russia, the migration distance was approximately 500 km. A natal origin in mountain areas (the Alps, Carpathian Mountains, Caucasus) could be possible based on the δ^2^H_n wing_ values (Fig. 3a). In case of movements over open water (i.e. the Baltic Sea), Population 1 could originate from regions in Norway, Sweden and Finland (Fig. 3a). By contrast, none of these regions was a likely origin of Population 2 (Fig. 3b). Instead, the natal origin of Population 2 stretched from the southernmost part of Norway over the northern part of the Mediterranean to Kazakhstan in the East (Fig. 3b). Notably, Population 2 could originate from the surroundings of the capture location in Estonia as well (Fig. 3b).

## Discussion

Our findings revealed that part of *A. mixta* individuals sampled at a bird observatory in Estonia originated from regions north or east of the study site, providing evidence for southbound autumn migration of dragonflies along the Baltic coast. Contrary to our initial hypothesis, actively migrating *A. mixta* individuals were predominantly caught under headwind conditions, potentially forcing them to remain within the flight boundary layer where the Heligoland traps were positioned. Consistent with our expectation, a larger proportion of males than females of *A. mixta* engaged in migration along the Baltic coast during autumn. However, further research is needed to investigate the underlying factors.

### Southbound migration of *A. mixta* at the Baltic coast in autumn

As a prerequisite for the assignment of potential natal origins of *A. mixta*, we established a dragonfly wing isoscape based on the combination of δ^2^H_n wing_ values of *A. cyanea* collected in Europe with those of different species of Aeshnidae from North America. The regression parameters of the relationship between the H isotope signature of annual precipitation and dragonfly wings in our study (Fig. 2a) were very similar to those established by [11]. The dragonfly wing isoscape displayed discernible geographical patterns (Fig. 2b) that allowed for the assignment of natal origins in our study.

The sampled *A. mixta* individuals comprised two distinct populations with different potential natal origins. Population 2 could have migrated from southern directions or originate from the proximity of the capture location in Estonia (Fig. 3b). Migration from southern directions would result in an environmentally induced decreased survival and likely also a decreased reproductive habitat quality and would not represent an ecological incentive for northbound migration. Therefore, we infer a local origin potentially including individuals that were drifted by the prevailing southerly wind directions from proximate regions in Latvia. Thus, we have to partly reject our first hypothesis: A proportion of the sampled individuals of *A. mixta* did not migrate. On the one hand, assuming that the sampled individuals of Population 2 did not represent the entire local population, our finding might confirm the concept of partial migration [1]. On the other hand, it is plausible that Population 2 was captured at their migration origin and subsequently migrated to the South after the end of the sampling period. This scenario would imply a phenomenon referred to as “leap-frog” migration, observed in certain bird species where southern populations migrate later than their northern counterparts [44, 45].

By contrast, the potential natal origins of Population 1 (Fig. 3a) confirm a migratory behaviour of this population and thus, our first hypothesis. The actively migrating population of *A. mixta* in our study could have their natal origin in (i) southern/southeastern European mountain ranges, (ii) regions in Russia located northeast of the capture location or in (iii) Fennoscandia (Fig. 3a). Although the species range of *A. mixta* also covers mountain ranges [46], we consider a migration from these regions unlikely given the lack of ecological incentives for northbound migration in autumn (see above) and the fact that no actively migrating individual had its potential origin within the distance between the mountain ranges and the sampling spot. Migration from regions in Russia located northeast of our study site could apply but would imply migration distances of more than 500 km. Southern Fennoscandia could represent a likely yet less distant origin of the actively migrating population (Fig. 3a). For example, migration of *A. mixta* has been observed in Finland previously [47], and southern Finland is relatively close (ca. 250 km) to our study site although the Gulf of Finland might represent a migration barrier. However, migration across large water bodies have been observed for other dragonfly species [7, 48]. In general, the prevailing southerly direction of *A. mixta* migration from Russia or Finland towards Estonia, as observed in our study, aligns with findings from multiple studies on autumn insect migration [26, 49–51].

Notably, the region where most of the individuals of the actively migrating population had a probable natal origin (Fig. 3a) lies beyond the known area range of *A. mixta* [46]. This is in line with the observed northward range expansion of dragonflies [52] that can reach up to 300 km in ten years for *A. mixta* [46, 53, 54]. Therefore, our findings suggest that the northern range limit of *A. mixta* may lie further north (e.g., 64 °N) than previously believed.

The timing of the migration period in all of our study years was identical to that of [17] and of several sightings of *A. mixta* passing by bird observatories along the Baltic coast [17, 20, 22, 23]. The last individuals of *A. mixta* were caught by the end of September in Estonia (our study), in Latvia [17, 23], and in the Kaliningrad oblast (Rybachy) [20]. Our results are opposed to previous assumptions on massive and irruptive dragonfly migrations [24] but in line with a study along a coast in eastern North America [19]. A review of previous findings, combined with our results, thus suggest a regular southbound autumn migration of *A. mixta* along the Baltic coast.

### Wind conditions during migration

Headwinds (Southerlies) dominated at our study site in Estonia during the study periods in 2009, 2010 (Fig. 1c) and in 2015 (Fig. 1b). The average wind speed of 23 km h^− 1^ during the study period (Table S1) was in the range of the self-powered airspeed of 18 to 29 km h^− 1^ that dragonflies can reach [6, 50, 55]. Therefore, in our study, *A. mixta* likely migrated close to the ground within the flight-boundary layer where the insects’ airspeed is higher than the wind speed and where they can stick to their preferred direction even in headwinds [2, 49]. This might suggest that under headwind conditions, *A. mixta* stays close to the ground and is caught in the traps at bird observatories while the species makes use of tailwind conditions (Northerlies) at higher altitudes preventing capture. It is remarkable though that also under tailwind conditions captures of 20 to > 100 individuals of *A. mixta* per day were reported in a bird trap in Latvia [17] which is comparable to our Estonian study (< 18 to 135 *A. mixta* individuals per day) despite the energy-costly maintenance of high airspeed [50] under headwind conditions in the latter study. Contrary to our second hypothesis but in line with the migration behaviour of a moth species [49], our results highlight that *A. mixta* does not wait for favourable wind conditions and continues the southbound autumn migration even in case of headwinds.

### Characteristics of the actively migrating population

The characteristics of the actively migrating population might provide hints for the reason of migration. The actively migrating population of our study and of migrating *A. mixta* individuals in other studies [24] was dominated by males. Skewed sex ratios in favour of males were also observed for other migrating insect species [32, 56]. Brattström, Shapoval [32] argued that males of *Vanessa atalanta* move around more than females and thus, are more likely to be caught. If this was a systematic sampling artefact in our study it would apply to both populations which was not the case. It was speculated that sexes of a sub-population of *V*. *atalanta* might use different hibernation sites [32] and thus, different migration routes. However, the univoltine *A. mixta* does not hibernate as an imago. Furthermore, whether *A. mixta* – like other dragonfly and insect species [1, 2, 8, 57] – reproduces after migration followed by hatching of the next generation in the following spring at southern, more favourable destinations remains an open question. Therefore, we consider sex-specific migration routes not likely. Alternatively, the dominance of males might indicate the competition for resources (food, mates, territory). Territorial behaviour is known for males of *A. mixta* [58] and could force particularly males to avoid competition and to migrate which corroborates our third hypothesis. However, skewed sex ratios of actively migrating dragonfly populations in our single-year study requires further corroboration by repeated sampling in upcoming years, because sex ratios in migrating hoverflies were year-specific and levelled out over different years [59].

## Conclusions

We found a population-specific southbound autumn migration of *A. mixta* which continued under unfavourable headwind conditions at the bird observatory in Estonia. Taken together with other observations along the Baltic coast, we conclude that southbound autumn migration of *A. mixta* is a regular phenomenon. The actively migrating population was dominated by males. Further repeated, large-scale studies along the Baltic coast are necessary to pinpoint the migratory pattern namely partial versus leap-frog migration and the reason for migration of this species. Such studies should also comprise locations north of the known species range of *A. mixta* because of the rapid climate-change induced range expansion. Finally, the establishment of networks among bird observatories to collect inadvertently trapped insects would help to evaluate insect-mediated energy and nutrient transfer linking distant ecosystems [3, 10].

### Electronic supplementary material

Below is the link to the electronic supplementary material.

Supplementary Material 1

## Data Availability

The datasets supporting the conclusions of this article are included within the article and its additional files. The R code is also provided in the supplementary information.

## Abbreviations

δ^2^H_n wing_: H isotope signature of nonexchangeable H in dragonfly wings
δ^2^H_p_: H isotope signature of annual precipitation
