# Supplementary material for: Autumn migration of the migrant hawker (Aeshna mixta) at the Baltic coast
Source: Mov Ecol. 2023 Aug 24;11:52. doi: 10.1186/s40462-023-00415-z (PMC10464154; doi:10.1186/s40462-023-00415-z)
Supplement: Supplementary file 1 — Supplementary Material 1 [file 40462_2023_415_MOESM1_ESM.docx]

Supplementary information for

**Autumn migration of the migrant hawker (*Aeshna mixta*) at the Baltic coast**

Yvonne Oelmann^1^, Diana Fiedler^1^, Rune Michaelis^1,2^, Andreas Braun^3^, Meelis Leivits^4^,
Philipp Gschwind^1,5^, Harald Neidhardt^1^, Christoph Willigalla^6^

^1^ Geoecology, Department of Geosciences, University of Tübingen, 72070 Tübingen, Germany

^2^ Lower Saxon Wadden Sea National Park Authority, 26382 Wilhelmshaven, Germany

^3^ Geoinformatics, Department of Geosciences, University of Tübingen, 72070 Tübingen, Germany

^4^ Estonian Environment Agency, Nigula Nature Centre, 86107 Reinu village, Estonia

^5^ GÖG - Gruppe für ökologische Gutachten, 70599 Stuttgart, Germany

^6^ Willigalla Ökologische Gutachten, 55124 Mainz, Germany

***Correspondence to:** Yvonne Oelmann, yvonne.oelmann@uni-tuebingen.de

Christoph Willigalla, christoph@willigalla.de

**1. Study site**


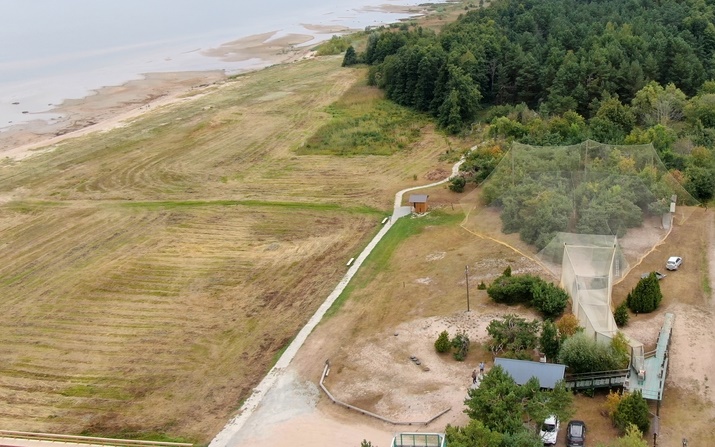
**Figure S1:** View from the South at the Helgoland trap (entrance size: height 17 m x width 51 m; 73 m long; size at end: 10 m x 10 m) at Kabli station, Estonia (58°0'51''N 24°26'58''E). Photo credit: Rein Nellis.

**2. The H isotope signature of the nonexchangeable fraction in dragonfly wings**

The δ^2^H values of the total H pool (δ^2^H_t_) in dragonfly wings are composed of a nonexchangeable (H_n_) fraction and of an exchangeable fraction (H_ex_) with respective δ^2^H_n_ and δ^2^H_ex_ values. δ^2^H values of the total H pool were measured directly. The δ^2^H_ex_ values were set to a known δ^2^H value via steam equilibration (see Section 3.) which clearly deviates from the natural abundance level. Assuming that H_n_ and H_ex_ are the only two H-pools (H_n_ + H_ex_ = 1), the fraction of H_n_ as well as its isotope signature in dragonfly wings (δ^2^H_n wing_) can be calculated by means of a mass balance approach (1). For further information about the steam equilibration and the calculation of δ^2^H_n_ values refer to Ruppenthal, Oelmann (1).

**3. Steam equilibration and isotope analysis of dragonfly wing samples**

To account for the exchangeable fraction of H (H_ex_) that does not carry a robust isotope signal of the wings we used the equilibration device based on the design of Wassenaar and Hobson (2) and modified by Ruppenthal, Oelmann (1). This setup consists of a stainless-steel vacuum vessel which is connected to a vacuum pump at the one end and to an argon bottle at the other end. Samples were placed in the steel vessel and the lids were screwed tight. After evacuating the vessel for one hour, the vessel was disconnected from the vacuum line and 3.5 ml of the equilibration water (δ^2^H values of
-268, -75, and +113 ‰ VSMOW, respectively, in separate runs) was injected with a syringe through a silicone rubber septum. Subsequently, the vessel was placed in a fan-assisted heating oven at 120 °C overnight. This time is necessary to reach the isotopic equilibrium between the exchangeable sample H and the injected equilibrium water H (1). The next day, the vessel was reconnected to the vacuum line and evacuated for 1.5 h. To depressurize the vessel to ambient air pressure, the vessel was flooded with argon gas (purity grade 5.0) which guarantees that the samples do not re-equilibrate with ambient air. While continuously flushing the vessel with the argon gas, the equilibrated samples were taken out of the vessel and directly sealed airtight with end cutting pliers. This general approach guarantees effective replacement of all isotopically exchangeable sample H with the equilibrium water H (1). Samples were pyrolysed in the EA reactor at 1450 °C. The glassy carbon tube of the EA was packed with glassy carbon granules. Tin capsules did not show a measurable H_2_ blank and the correction factor for H_3_^+^ ions produced in the ion source was ascertained before each carousel run using the automated procedure of the IRMS software (Ion Vantage, Elementar, Germany). Over the measurement period, the H_3_^+^ correction factor varied between 7.2 and 7.8 ppm nA^-1^. For normalisation of measured δ^2^H values of each carousel run, three international laboratory standards (VSMOW, USGS46 and GISP) were used. The reproducibility of an internal laboratory standard was ± 1.7 ‰ (n = 30).

**4. Assignment of natal origins of individuals and populations**

The precipitation isoscape was imported to R Studio, version 4.0.3 (3) and converted into a data frame containing the columns ‘x’ and ‘y’ representing the coordinates and the δ^2^H_p_ value at the corresponding coordinates, respectively. This was achieved using the R packages raster, rgdal and sp. To convert the precipitation isoscape into a wing isoscape, the linear regression equation (cf. Fig. 2a in main text) was applied to each δ^2^H_p_ grid value of the precipitation isoscape. To proceed, the file had to be saved as a point shapefile and then imported into QGIS. Using the Rasterize tool, the shapefile was converted to a raster. The values calculated for the dragonfly δ^2^H model isoscape were selected as raster cell value, output raster size units were set to ‘georeferenced units’ representing a horizontal and vertical resolution of 0.083333° (corresponding to 5 angular minutes), which matches the pixel size of the precipitation isoscape raster.

To obtain the potential natal origin of dragonflies, the wings of 88 specimens of *Aeshna mixta* were used. The assignment of the dragonfly populations to their origin was conducted based on probability-based geostatistical assignment methods described by Hobson, Soto (4), Hobson, Anderson (5), Hobson, Doward (6), Hobson, Jinguji (7) and Wunder (8). As only one isotope value per grid point is given by the isoscape, an empirically based stochastic component is combined with a calibrated isoscape for the assignment (8).

The natal origins of the dragonflies were calculated using the data frame of the dragonfly δ^2^H model isoscape (Fig. 2 in the main text), the 15.1‰ residual standard deviation from the regression equation and the respective δ^2^H_n wing_ value of every individual. Using these values and the ‘dnorm’ function of the R package ‘stats’, the density of the normal distribution for each pixel within the wing isoscape was determined for every individual, thus the following procedure was conducted 88 times. The calculated values were stored in a new column. To estimate the probability of the origin of every dragonfly, a probability value was calculated for each grid point of the isoscape. This was done by dividing the density of the normal distribution value of each pixel by the sum of all density of the normal distribution values (4, 5). Thus, each grid value now represented an estimated probability of being a potential origin of the respective dragonfly. The ascertained probability values for each individual pixel were now stored in a new column. The calculations for each individual can be found in the provided R script. After the import into QGIS, the .csv files were converted to raster files as previously specified (see 4. Establishment of an isoscape applicable to Europe). The column containing the calculated probability values was specified as the raster cell value and every probability map was exported as a .tif file. Subsequently, the probability maps for each individual were first rescaled (Raster Analysis -> rescale Raster, range: 0 - 100) and the rescaled raster was then classified via QGIS GRASS using the tool ‘r.reclass’ (classification rule: 0 thru 66 = 0, * = 1). Thus, a probability of ≥ 66% indicated a probable origin (binary pixel value = 1), whereas probabilities of < 66% were excluded as probable origins (binary pixel value = 0) of the respective individual. The 88 resulting binary probability maps were assigned to the two identified populations using the IDs of the individuals and then added up per population. This resulted in two maps, one for each population, showing the number of individuals in the population potentially originating from the pixel (see Fig. 2).

**5. References**

1. Ruppenthal M, Oelmann Y, Wilcke W. Isotope ratios of nonexchangeable hydrogen in soils from different climate zones. Geoderma. 2010;155(3-4):231-41.

2. Wassenaar LI, Hobson KA. Improved method for determining the stable-hydrogen isotopic composition (delta D) of complex organic materials of environmental interest. Environ Sci Technol. 2000;34(11):2354-60.

3. R Core Team. R: A language and environment for statistical computing. R Foundation for Statistical Computing, Vienna, Austria Available online at <https://wwwR-projectorg/>. 2020.

4. Hobson KA, Soto DX, Paulson DR, Wassenaar LI, Matthews JH. A dragonfly (d2H) isoscape for North America: a new tool for determining natal origins of migratory aquatic emergent insects. Methods Ecol Evol. 2012;3(4):766-72.

5. Hobson KA, Anderson RC, Soto DX, Wassenaar LI. Isotopic evidence that dragonflies (*Pantala flavescens*) migrating through the Maldives come from the northern Indian subcontinent. Plos One. 2012;7(12).

6. Hobson KA, Doward K, Kardynal KJ, McNeil JN. Inferring origins of migrating insects using isoscapes: a case study using the true armyworm, Mythimna unipuncta, in North America. Ecol Entomol. 2018;43(3):332-41.

7. Hobson KA, Jinguji H, Ichikawa Y, Kusack JW, Anderson RC. Long-distance migration of the Globe Skimmer dragonfly to Japan revealed using stable hydrogen (delta H-2) isotopes. Environmental Entomology. 2021;50(1):247-55.

8. Wunder MB. Using Isoscapes to Model Probability Surfaces for Determining Geographic Origins. West JB, Bowen GJ, Dawson TE, Tu KP, editors. New York: Springer; 2010. 251-70 p.

**6. R code**

**# required packages**

library(readxl)

library(raster)

library(sp)

library(rgdal)

library(tidyverse)

# load "data" folder into working directory (use "getwd()" to check working directory)

# required datasets in folder: Isoscape (isoscape.tif), Data on wing δ2H values of the dragonflies (d2H_A_mixta.xlsx)

**# Population distinction via k-means clustering**

# import the spreadsheet containing wing δ2H values of the A. mixta individuals

d2H_A_mixta <- read_excel("~/data/d2H_A_mixta.xlsx")

colnames(d2H_A_mixta) = "delta2H"

km <- kmeans(d2H_A_mixta,centers=2)

d2H_A_mixta$Population <- as.factor(km$cluster)

# plot

kmeansclustering <- ggplot(d2H_A_mixta, aes(x=delta2H)) +

geom_histogram(aes(fill=Population,y=..count..),

binwidth=2, color="grey50")+

xlab(expression(delta^2*"H"*""[n] ("‰")))+

ylab("n individuals")+

theme(panel.background = element_blank(), axis.line = element_line(colour = "black"))

kmeansclustering

**# processing of the isoscape raster data**

# import of the isoscape (.tif) and conversion into a data frame with columns "x" and "y" for the coordinates and the respective precipitation δ2H value of at the corresponding coordinates.

isoscape <- raster("~/data/isoscape.tif")

df_isoscape <- as.data.frame(rasterToPoints(isoscape))

# add columns "Wing", "Dnorm" and "Probability"

df_iso_gw <- df_isoscape %>% add_column(Wing = NA)

df_iso_Dn <- df_iso_gw %>% add_column(Dnorm = NA)

df_iso_Pr <- df_iso_Dn %>% add_column(Probability = NA)

# change data type of added columns

df_iso_Pr[sapply(df_iso_Pr, is.logical)] <-lapply(df_iso_Pr[sapply(df_iso_Pr, is.logical)], as.double)

# check data type, must be "num"

str(df_iso_Pr)

Isoscape <- df_iso_Pr

**# wing isoscape calculation**

Isoscape[,"Wing"] <- Isoscape[,"isoscape"]*0.93-38.33 #slope and intercept of regression equation (see Fig. S2)

write.csv(Isoscape,"wing_isoscape.csv") #save as .csv file, add to "data" folder

**# probability calculation**

# Import wing_isoscape.csv, change column data type to double ("num")

# exemplary on the basis of one individual (Lab Code 43768)

Isoscape_wing <- read_csv("~/data/wing_isoscape.csv")

Isoscape_wing <- as.data.frame(Isoscape_wing)

Isoscape_wing[sapply(Isoscape_wing, is.logical)] <-lapply(Isoscape_wing[sapply(Isoscape_wing, is.logical)], as.double)

# calculate the density of the normal distribution for each pixel within the wing isoscape

Isoscape_wing[,"Dnorm"] <- dnorm(-116.77, Isoscape[,"Wing"], 15.1) #dnorm(respective wing δ2H value, Isoscape[,"Wing"], SD of regression equation)

# calculate probability of origin by dividing the density of the normal distribution value of each pixel by the sum of all density of the normal distribution values

Isoscape_wing[,"Probability"] <- Isoscape_wing[,"Dnorm"]/((sum(Isoscape_wing[,"Dnorm"])))

write.csv(Isoscape_wing,"probability_respective_individual.csv")

**7. Weather conditions** **during the study**

**Table S1:** Weather conditions (mean temperature and wind speed during the daily capture periods from 8 am to 7 pm) in the study periods in 2009, 2010 and 2015. Note that the 2015 data were obtained online (Data from MET Norway; https://www.yr.no/en/details/table/2-591805/Estonia/P%C3%A4rnumaa/P%C3%A4rnu%20linn/Kabli) and refer to the daily average.

| **Date** | **Temperature**  **[° C]** | **Wind speed**  **[km h^-1^]** |
| --- | --- | --- |
| 22.08.2009 | 19.0 | 33.7 |
| 23.08.2009 | 16.0 | 33.7 |
| 24.08.2009 | 19.5 | 33.7 |
| 25.08.2009 | 19.0 | 33.7 |
| 26.08.2009 | 20.7 | 33.7 |
| 27.08.2009 | 18.4 | 47.8 |
| 28.08.2009 | 20.3 | 13.9 |
| 29.08.2009 | 18.4 | 19.7 |
| 30.08.2009 | 17.3 | 98.1 |
| 31.08.2009 | 17.5 | 68.1 |
| 01.09.2009 | 20.3 | 13.5 |
| 02.09.2009 | 23.5 | 34.8 |
| 03.09.2009 | 21.0 | 9.1 |
| 04.09.2009 | 19.3 | 36.2 |
| 05.09.2009 | 18.4 | 5.8 |
| 06.09.2009 | 18.0 | 35.3 |
| 07.09.2009 | 17.9 | 66.7 |
| 08.09.2009 | 18.3 | 44.1 |
| 09.09.2009 | 22.0 | 46.9 |
| 10.09.2009 | 20.5 | 16.9 |
| 11.09.2009 | 20.7 | 22.5 |
| 12.09.2009 | 18.8 | 11.5 |
| 13.09.2009 | 20.5 | 12.8 |
| 15.09.2009 | 18.0 | 15.1 |
| 16.09.2009 | 17.1 | 18.9 |
| 17.09.2009 | 16.8 | 62.2 |
| 18.09.2009 | 16.8 | 43.5 |
| 19.09.2009 | 17.5 | 42.7 |
| 20.09.2009 | 18.0 | 41.8 |
| 21.09.2009 | 16.8 | 76.0 |
| 22.09.2009 | 16.0 | 48.7 |
| 23.09.2009 | 15.0 | 75.9 |
| 24.09.2009 | 14.0 | 92.3 |
| 25.09.2009 | 14.3 | 40.3 |
| 02.10.2009 | 16.0 | 49.9 |
| 10.10.2009 | 14.0 | 8.5 |
|  |  |  |
| 18.08.2010  20.08.2010  21.08.2010  22.08.2010  23.08.2010  24.08.2010  25.08.2010  26.08.2010  27.08.2010  28.08.2010  29.08.2010  30.08.2010  31.08.2010  01.09.2010  05.09.2010  06.09.2010  07.09.2010  08.09.2010  09.09.2010  10.09.2010  11.09.2010  12.09.2010  13.09.2010  14.09.2010  15.09.2010  16.09.2010  17.09.2010  18.09.2010  20.09.2010  21.09.2010  23.09.2010  24.09.2010  25.09.2010  26.09.2010  29.09.2010  30.09.2010  01.10.2010  10.10.2010 | 20.0  20.8  19.0  19.9  19.6  20.8  18.7  18.0  20.8  20.6  17.0  15.0  21.7  16.5  15.0  17.0  19.8  15.8  21.0  15.7  14.0  15.1  20.0  17.8  14.0  14.4  13.0  14.0  14.3  12.0  14.5  17.4  19.1  17.0  11.0  9.0  11.5  7.0 | 8.5  11.4  68.5  63.9  77.5  43.0  54.2  68.1  8.2  5.0  8.5  0.0  8.2  7.2  8.5  139.5  0.5  2.9  13.1  11.4  3.0  18.5  24.2  49.5  57.4  57.6  81.5  84.0  39.0  72.0  67.0  25.6  18.1  25.0  48.7  15.6  8.5  15.6 |
|  |  |  |
| 27.08.2015  28.08.2015  29.08.2015  30.08.2015  31.08.2015  01.09.2015  02.09.2015  03.09.2015  04.09.2015  05.09.2015  06.09.2015 | 18.9  18.0  17.5  16.6  16.0  14.6  14.1  16.7  14.9  15.2  14.5 | 19.1  30.3  35.7  26.4  12.6  24.6  20.4  27.8  21.1  26.9  19.5 |

**8. Phenology** **of the migration of *A. mixta* in the study years**

**Figure S2:** Phenology of the migration of *A. mixta* in the study periods in 2009, 2010 and 2015.
